# Evaluation of mindfulness-based cognitive therapy for life and a cognitive behavioural therapy stress-management workshop to improve healthcare staff stress: study protocol for two randomised controlled trials

**DOI:** 10.1186/s13063-018-2547-1

**Published:** 2018-04-02

**Authors:** Clara Strauss, Jenny Gu, Nikki Pitman, Cavita Chapman, Willem Kuyken, Adrian Whittington

**Affiliations:** 1 School of Psychology, University of Sussex, Pevensey Building, Falmer, BN1 9QH UK; 2 Sussex Partnership NHS Foundation Trust, R&D Department, Sussex Education Centre, Nevill Avenue, Hove, BN3 7HZ UK; 3 Health Education England Kent, Surrey and Sussex, Crawley, West Sussex UK; 4 Department of Psychiatry, University of Oxford, Warneford Hospital, Headington, Oxford, OX3 7JX UK

**Keywords:** RCT, Mindfulness, MBCT, MBCT-L, Cognitive behavioural therapy, CBT, NHS, Workplace, Stress, Healthcare staff, Healthcare professional, Mental health, Sickness absence, Compassion, Wellbeing, Burnout

## Abstract

**Background:**

Healthcare workers experience higher levels of work-related stress and higher rates of sickness absence than workers in other sectors. Psychological approaches have potential in providing healthcare workers with the knowledge and skills to recognise stress and to manage stress effectively. The strongest evidence for effectiveness in reducing stress in the workplace is for stress-management courses based on cognitive behavioural therapy (CBT) principles and mindfulness-based interventions (MBIs). However, research examining effects of these interventions on sickness absence (an objective indicator of stress) and compassion for others (an indicator of patient care) is limited, as is research on brief CBT stress-management courses (which may be more widely accessible) and on MBIs adapted for workplace settings.

**Methods/design:**

This protocol is for two randomised controlled trials with participant preference between the two trials and 1:1 allocation to intervention or wait-list within the preferred choice. The first trial is examining a one-day CBT stress-management workshop and the second trial an 8-session Mindfulness-Based Cognitive Therapy for Life (MBCT-L) course, with both trials comparing intervention to wait-list. The primary outcome for both trials is stress post-intervention with secondary outcomes being sickness absence, compassion for others, depression symptoms, anxiety symptoms, wellbeing, work-related burnout, self-compassion, presenteeism, and mindfulness (MBCT-L only). Both trials aim to recruit 234 staff working in the National Health Service in the UK.

**Discussion:**

This trial will examine whether a one-day CBT stress-management workshop and an 8-session MBCT-L course are effective at reducing healthcare staff stress and other mental health outcomes compared to wait-list, and, whether these interventions are effective at reducing sickness absence and presenteeism and at enhancing wellbeing, self-compassion, mindfulness and compassion for others. Findings will help inform approaches offered to reduce healthcare staff stress and other key variables. A note of caution is that individual-level approaches should only be part of the solution to reducing healthcare staff stress within a broader focus on organisational-level interventions and support.

**Trial registration:**

ISRCTN Registry, ISRCTN11723441. Registered on 16 June 2017.

Protocol Version 1: 24 April 2017.

Trial Sponsor: Sussex Partnership NHS Foundation Trust (ResearchGovernance@sussexpartnership.nhs.uk).

**Electronic supplementary material:**

The online version of this article (10.1186/s13063-018-2547-1) contains supplementary material, which is available to authorized users.

## Background

Healthcare workers experience disproportionately high levels of work-related stress. A recent survey in the USA found that stress was higher in healthcare workers than in any other industry, with 69% of staff reporting feeling stressed and 17% reporting high levels of stress [1]. The picture is similar elsewhere. In the UK, for example, 37% of staff working in the National Health Service (NHS) report feeling unwell due to work-related stress [30] and NHS staff are more likely to experience work-related stress compared to staff from any other public sector profession, with 61% feeling stressed all or most of the time [20]. Sickness absence is also highest in the NHS out of all the large public sector organisations [31]. In addition to the serious personal and economic consequences, high levels of stress in healthcare staff may negatively impact on patient care and safety [18]. There is therefore a need to find effective ways of reducing healthcare staff stress and interventions based on psychological theory and related psychological therapeutic interventions provide one potential solution.

It is first helpful to clarify what we mean by stress. The transactional theory of stress has arguably been most influential in recent decades [25, 26]. This suggests that stress is neither a property of situations and nor is it a property of the person. Rather, stress occurs as a transaction, or interaction between the situation and the person and arises as a consequence of the person’s appraisal of the situation. The primary appraisal concerns whether the situation is perceived as a threat to the person, and the secondary appraisal concerns whether the person perceives they have the resources (including personal resources) to cope with the threat [26]. This theory has been applied to a wide variety of contexts, including the workplace [24]. The transactional theory of stress is potentially helpful in empowering people to identify stress-related appraisals and choosing how best to respond, even in the context of highly demanding workplace situations. Cognitive behavioural therapy (CBT) and Mindfulness-Based Cognitive Therapy (MBCT) are two psychological approaches that could facilitate increased awareness of stress-related (and other) appraisals, re-evaluation or non-judgmental acceptance of these appraisals and greater awareness of choices available of how best to respond.

A meta-review found that stress-management interventions based on CBT have the strongest evidence for effectiveness in reducing work-related stress [21]. CBT stress-management involves identifying how thoughts (including appraisals), feelings, behaviours and physical sensations interact to contribute to stress, and using this information to identify strategies for preventing or reducing stress. Strategies may include identifying and re-evaluating the accuracy of stress-related thoughts (appraisals), identifying behaviours that contribute to stress and choosing alternative, more helpful behaviours, and identifying strategies to reduce physiological arousal associated with stress. By intervening in this way, the stress-related maintenance cycle between thoughts, feelings, behaviours and physical sensations can be broken and replaced with a stress-relieving maintenance cycle.

There is also growing evidence that mindfulness-based interventions (MBIs) improve stress in healthcare staff [9, 12, 14, 37, 41], including in NHS settings [28]. Mindfulness is characterised by non-judgemental awareness and acceptance of present-moment experiences (thoughts, feelings, sensations etc.), and greater awareness of helpful behavioural choices available. Mindfulness-based cognitive therapy (MBCT) [35] integrates aspects of CBT within an MBI and was originally developed to prevent depressive relapse, for which it has well-established benefits [23]. A recent adaptation for non-clinical populations that draws on the same structure and techniques as MBCT is MBCT for life (MBCT-L) [5]. Adaptations within MBCT-L include a greater focus on wellbeing, appreciation and gratitude, making this better suited in workplace settings.

In summary, there is good evidence that CBT stress-management interventions and MBIs are effective at reducing work-related stress. However, there some important gaps in the current literature.

One gap is establishing if benefits extend to reduced sickness absence. Sickness absence is estimated to cost the NHS 2.5% of its entire budget [33]. This reduces the budget available for patient care whilst placing an additional strain on staff to cover the duties of the absent member of staff, which in turn may lead to increased levels of stress and greater risk of sickness absence for staff remaining at work. Randomised controlled trial (RCT) evidence is lacking examining the potential of CBT stress-management and MBIs to reduce sickness absence.

A second gap is in investigating if the benefits of these interventions on healthcare staff stress extend to variables associated with improved patient care. There is evidence from cross-sectional studies that healthcare staff stress is associated with compromised patient safety [18], but evidence is limited as to whether interventions such as CBT stress-management and MBIs might have a causal effect on indicators of patient care such as the capacity for compassion for others. Compassion has been defined as a multi-faceted capacity involving the ability to recognise suffering, understand the universality of human suffering, feel for the person suffering, tolerate uncomfortable feelings, and the motivation to act/acting to alleviate suffering [39]. Both CBT stress-management and MBCT-L might have an effect on compassion for others (including patients) by increasing awareness of present-moment thoughts, feelings and physical reactions, cultivating understanding of human suffering as universal (as both approaches conceptualise distress using universal psychological frameworks) and increasing awareness of choices available to act to alleviate suffering.

A third gap is evidence for brief CBT stress-management interventions [21] that may be more readily accessible to healthcare staff in increasingly demanding healthcare settings. Whilst we would advocate giving sufficient time for staff to attend to their own wellbeing, we also acknowledge that many healthcare staff would struggle to attend interventions over several sessions. However, brief CBT stress-management may not be effective in comparison to their longer counterpart interventions [21] and research is needed to assess effectiveness.

Finally, a fourth gap concerns the evidence for MBCT-L. This is a newly developed intervention, designed for non-clinical settings, but we cannot assume that the benefits of MBCT for preventing depressive relapse [23] will extend to MBCT-L reducing stress in healthcare staff.

This protocol is for two RCTs examining the effectiveness of two interventions for staff working in the NHS: (1) MBCT-L and (2) CBT-based stress management. Staff will select one of these two interventions and then will be randomly assigned to either the intervention arm or to the wait-list arm within their preferred choice. Our intention in offering staff a choice between these two interventions is to increase accessibility and choice, acknowledging that no one intervention is likely to meet the needs of all staff.

Given the existing evidence for these interventions is predominantly in stress-reduction, the primary hypothesis is that both interventions will be more effective than the wait-list at reducing stress post-intervention. Secondary hypotheses are that both interventions will be more effective than the wait-list post-intervention in: (1) reducing sickness absence; (2) improving compassion for others; (3) reducing anxiety symptoms; (4) reducing depression symptoms; (5) reducing work-related burnout; (6) improving compassion for self; (7) improving wellbeing; (8) reducing presenteeism and (9) improving mindfulness (in MBCT-L participants only). We also plan to explore participants’ experiences of their chosen intervention using thematic analysis of semi-structured interviews.

## Methods/design

### Design and sample size

This protocol is for a study of two superiority RCTs with participant preference between the two interventions with 1:1 allocation to either intervention or wait-list within the preferred choice. The randomisation procedure will be web-based and automated; the allocation sequence will be generated and participants randomised using block randomisation by Qualtrics (www.qualtrics.com), the online survey software. Members of the research team involved in the day-to-day management of the study will be blind to block size. Assessments will be completed online by participants at baseline and post-intervention, independent from members of the research team, to reduce risk of bias associated with researcher-administered assessments.

Sample size calculations were conducted using G*Power [13]. The study aims to have 140 participants giving complete data sets at baseline and post-intervention within each part of the study (i.e. aiming for 140 MBCT-L and 140 CBT study completers). Given the pressures on staff time and the online data collection method, it is conservatively assumed that 40% of participants will fail to complete measures post-intervention. This means that we aim to recruit 234 participants into each part of the study.

Sample size calculations are based on an estimated medium between-group effect on post-intervention stress outcomes (Cohen’s *d* = .50) between the intervention and wait-list arms with 90% power and *p* = .05. For MBCT-L, the estimated medium effect size is based on between-group post-intervention effects on stress reported in previous trials of MBIs for healthcare staff [9, 12, 14]. For the CBT stress-management workshop it was not possible to estimate the effect directly based on published trials as previous research has evaluated CBT stress-management interventions running over several sessions. We therefore assume that the effect size will be smaller in the current study than the large effect reported in a meta-analysis of multiple-session CBT stress-management interventions in the workplace [34] and therefore estimate a medium effect size.

Ten participants from the intervention arm of each RCT, who provide complete data sets at baseline and post-intervention and who complete their allocated intervention, will be interviewed about their experiences of their intervention. The sample size for the qualitative interviews is based on recommendations for thematic analysis from Braun and Clarke [7].

### Participants

Participants will be members of staff working in one of four NHS Trusts in the South of England (three mental health Trusts and one community Trust) with each Trust employing between 2500 and 5000 members of staff. Inclusion criteria are that participants (1) are employed by (or working in an honorary/voluntary capacity for) one of the four NHS trusts, (2) are currently in work (i.e. not currently on sickness absence), (3) have sufficient English language ability to understand intervention information and questionnaire content and (4) are adults (aged 18 years or older). There are no exclusion criteria.

### Procedure

**Fig. 1:**
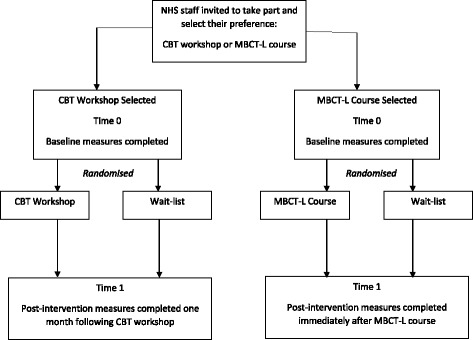
CONSORT flow diagram showing participant flow through the study. NHS, National Health Service; CBT, cognitive behavioural therapy; MBCT-L, mindfulness-based cognitive therapy for life

Recruitment is planned to take place between July and December 2017. The Consolidated Standards of Reporting Trials (CONSORT) diagram showing participant flow through the study is shown in Fig. 1. The study will be advertised to all Trust staff through adverts placed on intranets and staff bulletins. In addition, information about the study will be emailed to all staff. Participants consenting to take part in the study will first choose their preferred intervention. Staff can take part in one of the two studies, but not both. This will be checked to ensure that all consenting participants are enrolled in one of the two studies only. Following consent, participants will be sent a standardised e-mail by the research team containing a link to the baseline assessment measures hosted on Qualtrics based on their selected intervention (Time 0). Upon completion of baseline measures, participants will be randomised to their preferred intervention or to the wait-list for their preferred intervention. They will be sent a standardised e-mail informing them of their allocation and details of their intervention. After participants have completed their intervention or wait-list time period, they will be sent a standardised e-mail asking them to complete post-intervention measures online (Time 1). MBCT-L participants will be sent the link to post-intervention measures immediately after completion of the intervention. CBT participants will be invited to complete post-intervention measures one month after workshop completion. The research team will not be present for any of the online assessments (at baseline and post-intervention); measures will be completed by participants online and in their own time. To promote study retention, where necessary participants will be emailed at weekly intervals for up to 4 weeks with a reminder to complete their post-intervention assessment. Potential errors with data entry will be minimised as data will be entered by participants online.

Ten participants from each intervention arm, who complete both baseline and post-intervention measures, will be invited to take part in an optional phone interview about their experiences of their preferred intervention.

### Interventions

#### Mindfulness-based cognitive therapy for life

MBCT-L [5] is an adaptation of MBCT originally developed by Segal, Williams and Teasdale [35, 36]. MBCT was originally developed for people with a history of recurrent depression at risk of depressive relapse and integrates CBT strategies with mindfulness practice and inquiry about practice. MBCT-L was developed to be applicable to the general population across the spectrum of wellbeing and draws on the same structure and techniques as MBCT. MBCT-L is an 8-week group intervention (with a pre-course orientation session) where participants are guided in mindfulness practice and engage with a range of CBT strategies. In this study, each group will be led by one or two MBCT teachers and will consist of up to 15 participants. Each session will take 2 h and participants will be invited to complete approximately 40 min per day of mindfulness practice and other home tasks. Content in the sessions will include guided mindfulness practices, inquiry into experiences following practices, weekly homework review, and teaching/discussion of CBT skills. The teachers leading the groups will have completed MBCT teacher training and will meet MBCT teacher criteria set out by the UK Network of Mindfulness-Based Teacher Training Organisations. Teachers will have completed additional MBCT-L training. Supervision will be provided on at least three occasions per group by a teacher who meets the supervisor criteria set out by the UK Network of Mindfulness-Based Teacher Training Organisations and who has attended 2-day MBCT-L training. Intervention completion is defined as attending at least four of the eight sessions.

#### Cognitive behavioural therapy

This 1-day (6 h) workshop will teach participants CBT approaches to managing work-related stress. The workshop is divided into three broad sections. The first part of the workshop introduces a CBT formulation of work-related stress, drawing on the transactional theory of stress [25, 26] and the CBT maintenance cycle highlighting the inter-relationships between thoughts, feelings, physical sensations and behaviours, within work (and other) contexts. Participants will have the opportunity to formulate their own work-related stress experiences within these frameworks. The second part of the workshop overviews strategies to intervene in the maintenance cycle, with a particular focus on cognitive and behavioural strategies. The third part of the workshop encourages participants to identify specific, measurable, attainable, relevant and timely (SMART) goals, drawing on learning from the workshop and making a commitment between participants to progress towards these goals. Each workshop will consist of up to 20 participants and will be facilitated by two mental health practitioners, who demonstrate the following skills and experience between them: (1) a qualified CBT therapist or practitioner psychologist who works with CBT as their primary therapeutic model; (2) experience of facilitating therapeutic workshops or groups and (3) a senior grade within their NHS trust or a registered mental health professional with significant experience working in their NHS trust. All facilitators will attend a 1-day training event led by a clinical psychologist and CBT therapist who developed the workshop materials and will receive at least one telephone consultation session. Intervention completion is defined as attending the whole of the 1-day workshop.

## Measures

### Primary outcome

#### Stress

The primary outcome measure will be the 7-item stress subscale from the 21-item short version of the Depression, Anxiety, and Stress Scales (DASS-21) [27]. The stress subscale of the DASS-21 measures the severity of core symptoms associated with stress. Participants are asked to indicate the presence of each symptom over the past week. Responses are given on a 4-point Likert scale, ranging from 0 (never) to 3 (almost always). The DASS-21 stress subscale has been found to have good internal consistency and convergent and discriminant validity [2, 19].

### Secondary outcomes

#### Sickness absence

Sickness absence data will be obtained from Human Resources departments in the NHS trusts. This will be recorded as the number of sickness absence days taken in the month following the end of the intervention period. Equivalent data from the same 1-month period in the previous calendar year will be obtained as a baseline measure for each participant. Reasons for sickness absence will not be recorded. This is to respect participant confidentiality.

#### Compassion for others

This will be measured using the Compassion for Others Scale (Gu, Baer, Kuyken, Cavanagh & Strauss: Developing and Validating New Self-Report Measures of Compassion: Compassion for the Self Scale (CSS) and Compassion for Others Scale (COS), in preparation), developed based on the empirically supported five-element definition of compassion as consisting of the ability to recognise suffering, understand the universality of human suffering, feel for the person suffering, tolerate uncomfortable feeling and the motivation to act/acting to alleviate suffering [16, 39]. Participants are instructed to indicate how true each statement is of them using a 5-point Likert scale, ranging from 1 (not at all true of me) to 5 (always true of me).

#### Depression

This will be measured using the depression subscale from the short version of the DASS-21 [27]. The depression subscale of the DASS-21 measures the severity of core symptoms associated with depression. Participants are asked to indicate the presence of each symptom over the past week. Responses are given on a 4-point Likert scale, ranging from 0 (never) to 3 (almost always). The DASS-21 depression subscale has been found to have good internal consistency and convergent and discriminant validity [2, 19].

#### Anxiety

This will be measured using the anxiety subscale from the short version of the DASS-21 [27]. The anxiety subscale of the DASS-21 measures the severity of core anxiety symptoms. Participants are asked to indicate the presence of each symptom over the past week. Responses are given on a 4-point Likert scale, ranging from 0 (never) to 3 (almost always). The DASS-21 anxiety subscale has been found to have good internal consistency and convergent and discriminant validity [2, 19].

#### Self-compassion

This will be measured using the Compassion for Self Scale (Gu, Baer, Kuyken, Cavanagh & Strauss: Developing and Validating New Self-Report Measures of Compassion: Compassion for the Self Scale (CSS) and Compassion for Others Scale (COS), in preparation), developed based on the empirically supported five-element definition of compassion as consisting of the ability to recognise suffering, understand the universality of human suffering, feel for the person suffering (in the case of self-compassion this would be the self), tolerate uncomfortable feelings and the motivation to act/acting to alleviate suffering [39]. Participants are instructed to indicate how true each statement is of them using a 5-point Likert scale, ranging from 1 (not at all true of me) to 5 (always true of me).

#### Wellbeing

Positive mental wellbeing will be measured using the 7-item Short Warwick Edinburgh Mental Wellbeing Scale (SWEMWBS) [38]. The SWEMWBS involves rating items on a 5-point Likert scale ranging from 1 (none of the time) to 5 (all of the time). Participants are asked to rate items based on their experience over the past 2 weeks. The SWEMWBS has been found to be highly correlated with the long version of the scale and good construct validity [38].

#### Burnout

This will be measured using the 22-item Maslach Burnout Inventory – Human Services Survey (MBI-HSS) [29]. The MBI-HSS was designed for professionals working in human services such as healthcare and consists of three distinct subscales, emotional exhaustion, depersonalisation, and personal accomplishment. Participants are asked about the frequency with which they have experiences related to the three subscales and items are answered on a 7-point Likert scale, ranging from 0 (never) to 6 (every day). The three subscales of the MBI-HSS has been found to have adequate internal consistency, test-retest reliability and convergent and discriminant validity.

#### Presenteeism

This will be measured using the 3 questions that assess presenteeism, from the Institute for Medical Technology Assessment Productivity Cost Questionnaire (iMTA PCQ) [6]. The overall iMTA PCQ is designed to assess and value productivity losses. The 3 presenteeism questions ask participants (1) if over the past 4 weeks, they worked whilst experiencing physical or psychological problems (yes/no) and if so, (2) how many days at work they were bothered by these problems and (3) how their performance on these days compared to their performance on normal working days. The third question is measured on a 10-point rating scale, ranging from 0 (on these days I could not do anything) to 10 (I was able to do just as much as I normally do). The first 2 questions originate from the short form of the Health and Labour Questionnaire (SF-HLQ) [40] and the third question from the Productivity and Disease Questionnaire (PRODISQ) [22]. The 3 questions have been found to have good test-retest reliability [6].

#### Mindfulness

This will be measured using the 15-item Five Facet Mindfulness Questionnaire (FFMQ-15) [4, 8, 17]. The FFMQ-15 is a short form of the 39-item FFMQ (FFMQ-39) and measures the general tendency to be mindful in everyday life. It includes the same five facets as the long form: observing, describing, acting with awareness, non-judging of inner experience and non-reactivity to inner experience. The factor structure of the FFMQ-15 is consistent with that of the FFMQ-39, there is strong correlation between the total facet scores of the short and long forms, and the two FFMQ versions do not differ significantly from each other in terms of convergent validity [17]. Previous research [3, 17, 42] found that in non-meditator samples, a four-factor hierarchical structure without the “observing” facet provided a superior fit compared to a five-factor hierarchical structure. As it is likely that our current sample has little or no previous meditation experience, “observing” items will be excluded from the total FFMQ-15 score. FFMQ-15 items are rated on a 5-point Likert scale, ranging from 1 (never or very rarely true) to 5 (very often or always true) and will be completed by participants randomised to MBCT-L or wait-list for MBCT-L only.

#### Intervention engagement – MBCT-L participants

MBCT-L participants will be asked to report the following post-intervention: (1) number of MBCT-L sessions attended, not including the orientation session (0–8); (2) average number of days per week engaged in a guided mindfulness practice, not including practice during the group session (0–7); (3) on days when practised, average number of minutes per day of mindfulness practice, not including practice during the group session; (4) ability to bring mindfulness principles into daily life (0–5); (5) ability to actively participate in MBCT-L sessions (0–5); (6) belief in effectiveness of mindfulness in helping to manage stressful situations (0–5); (7) difficulty in finding time to engage in between-session mindfulness practices; (8) satisfaction with the mindfulness teacher leading the course (0–5) and (9) levels of comfort with other group members (0–5). The 0–5 rating scales are all anchored by “not at all” (0) and “extremely” (5).

#### Intervention engagement – CBT participants

CBT workshop participants will be asked to report the following post-intervention: (1) attendance at CBT workshop (no, yes (part of the day), yes (whole day)); (2) satisfaction with the workshop facilitators (0–5) and (3) levels of comfort with other workshop members (0–5). The 0–5 rating scales are all anchored by “not at all” (0) and “extremely” (5).

All outcome measures, with the exception of sickness absence data, which will be requested from HR departments at the end of the study, will be administered at baseline and post-intervention. Demographic data (e.g., gender, age, ethnicity, marital status, education level) will be recorded at baseline only and engagement measures will be administered post-intervention only.

Ten participants from the intervention arm of each RCT, who provide complete data sets at baseline and post-intervention, will be interviewed about their experiences of their intervention by telephone, using an adapted version of the Change Interview [11]. A copy of the Change Interview published by Elliott and Rodgers can be found online [10]. This is a semi-structured interview designed to explore people’s experiences of psychological interventions, focusing on perceived helpful, unhelpful and missing aspects of the intervention. Each interview will take approximately 30 min, will take place over the phone, and will be audio recorded to aid transcription and data analysis.

### Planned data analysis

The intention in providing two interventions is to increase choice and accessibility. The intention is not to compare the effectiveness of the CBT intervention directly with the MBCT-L intervention and any such comparison would be problematic given the potential for selection bias (i.e. participants preferring MBCT-L may differ in a number of ways from participants preferring the CBT workshop). Intervention preference will be reported as the number and percentage of participants choosing each intervention type; however we would urge caution when interpreting these data as participant preferences may be driven by practicalities (e.g. location and dates of available courses) as much as by intervention preference.

Between-group differences at baseline on key demographic variables (age, gender, ethnicity, NHS trust, pay band and years working in the trust) and all outcome measures will be reported for each study. Findings will be reported for both intention-to-treat and per-protocol analyses. Hypotheses will be tested using mixed analysis of variance (ANOVA) for each intervention separately, with time (baseline, post-intervention) as the within-group variable and intervention arm (intervention, wait-list) as the between-group variable. Post-intervention between-group effect sizes (Cohen’s *d*) and 95% confidence intervals will be reported. Interaction effects will be followed up with within-group *t* tests, with Cohen’s *d* effect sizes and accompanying 95% confidence intervals for within-group change.

Qualitative data will be transcribed and thematic analysis will be performed in accordance with the Braun and Clarke [7] protocol. This will involve the researcher leading on the qualitative aspect of the study reading and re-reading transcripts, allocating codes to single units of meaning within each transcript, identifying sub-themes representing lower-order categories of meaning across participants (within CBT/MBCT-L separately) and finally identifying higher-order themes and the relationship between themes and sub-themes (for CBT/MBCT-L separately). This will be conducted under supervision from the lead author. Credibility will be checked through supervision and will be indicated through providing comprehensive extracts from participants to illustrate each theme and sub-theme.

### Dissemination

Findings will be written up for submission for publication in a peer-reviewed journal as 4 papers: (1) reporting on the quantitative findings from the RCT comparing MBCT-L with wait-list; (2) reporting on the quantitative findings from the RCT comparing the CBT-based stress management intervention with wait-list; (3) reporting on the qualitative findings of participating in MBCT-L and (4) reporting on the qualitative findings of participating in the CBT-based stress management intervention. A lay report of findings will be produced for dissemination to participants and other NHS Trust staff.

## Discussion

When compared to other professions, healthcare staff experience particularly high levels of work-related stress and sickness absence [1, 30, 31], with higher levels of stress associated with compromised patient care and safety [18]. Psychological approaches based on psychological theory of stress [25, 26] provide one solution. Evidence for reducing work-related stress is strongest for CBT stress-management [21] and mindfulness-based interventions [41]. However, effects on objective indicators of stress (such as sickness absence) and on factors associated with patient care (such as compassion for others) is largely unexplored. In addition, potential benefits of brief CBT-based stress management courses and of MBCT-L on healthcare staff levels of staff are unknown.

This is a protocol for two separate RCTs with participant preference examining the effects of two interventions, each compared to wait-list, for NHS staff. The first of these is a 1-day CBT stress-management workshop and the second is an 8-session MBCT for Life (MBCT-L) course. The primary outcome is stress, as this is the outcome with the greatest evidence of effects. Secondary outcomes include sickness absence, compassion for others, depressive symptoms, anxiety symptoms, compassion for self, work-related burnout, presenteeism and mindfulness (in MBCT-L participants). Effects on sickness absence would be of particular interest to healthcare employers and would provide an economic incentive to widen access to these interventions. Effects on compassion for others would suggest that benefits to staff might extend to improved patient care and this would lead to further research examining the effects of these interventions for staff on outcomes for their patients. Effects on wellbeing are also important to measure. Whilst the primary outcome in this study is stress, we are interested not only in the potential of the interventions to reduce stress and mental health symptoms, but also of the potential to enhance wellbeing and compassion for self and others. This focus is particularly highlighted in MBIs in the workplace [15] and in MBCT-L, with its emphasis on cultivating appreciation and gratitude [5].

A limitation of the design is that it will not be possible to directly compare outcomes between the two interventions as participants are not randomised between intervention types. A direct comparison of the two interventions could be explored in future trials, depending on the outcomes of the current study. Another limitation is that reasons for sickness absence will not be recorded in order to respect participants’ confidentiality. It is also possible that staff may be reluctant to disclose mental health reasons for sickness absence due to concerns about stigma, and that a physical health reason may be given instead. If the interventions have a beneficial effect on sickness absence due to poor mental health this should be reflected in an overall effect on sickness absence. There is also an important note of caution in relation to the interventions being evaluated. Providing psychologically informed interventions to healthcare staff as a means of reducing work-related stress could contribute to a culture whereby staff members are seen as solely responsible for managing their stress, absolving healthcare organisations from responsibility to provide supportive workplaces that do not place excessive demands on their staff. Individually targeted interventions such as CBT stress-management workshops and MBCT-L can be part of a solution to reducing work-related stress in the healthcare workplace. However, we suggest that this should occur in the context of organisational-level interventions to minimise stress, as these may play an additional important role in reducing stress in healthcare workplaces [32]. Within supportive healthcare organisations, our CBT stress-management workshop and MBCT-L have potential to provide staff with the skills to recognise signs of stress in themselves and the skills to act early to prevent stress from escalating, and thereby empowering staff to make choices about how they respond in stressful healthcare workplace settings.

## Trial status

At the time of manuscript submission, recruitment for this study was ongoing.

### SPIRIT guidelines

**Fig. 2:**
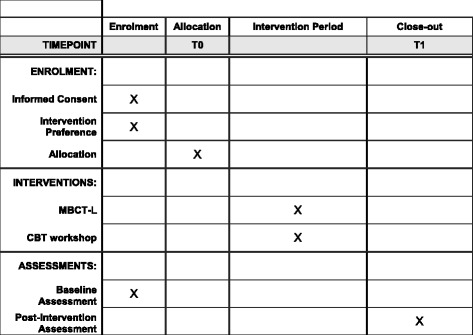
Schedule of enrolment, interventions, and assessments

Please see Fig. 2 for a copy of the Standard Protocol Items: Recommendations for Interventional Trials (SPIRIT) figure. The SPIRIT checklist can be found as Additional file 1.

## Additional file

Additional file 1: SPIRIT Checklist. (DOC 122 kb)

## Abbreviations

ANOVA: Analysis of variance
CBT: Cognitive behavioural therapy
DASS-21: Depression, Anxiety, and Stress Scales (short version)
FFMQ-15: Five Facet Mindfulness Questionnaire (15 item version)
FFMQ-39: Five Facet Mindfulness Questionnaire (39 item version)
HR: Human resources
iMTA PCQ: Institute for Medical Technology Assessment Productivity Cost Questionnaire
MBCT: Mindfulness-based cognitive therapy
MBCT-L: Mindfulness-based cognitive therapy for life
MBI: Mindfulness-based intervention
MBI-HSS: Maslach Burnout Inventory – Human Services Survey
NHS: National Health Service
RCT: Randomised controlled trial
SMART: Specific measurable achievable relevant time-limited
SWEMWBS: Short Warwick Edinburgh Mental Wellbeing Scale
